# Metagenomics-based analysis of microbial community dynamics and flavor compound correlations during rice-flavor Baijiu brewing

**DOI:** 10.3389/fbioe.2025.1638716

**Published:** 2025-08-05

**Authors:** Qun Li, Long Zhang, Rui Li, Jie Tang, Bin Lin, Chunyu Qin, Wei Jiang, Longxu An, Fan Zhang, Xingxing Shi, Shengzhi Yang, Qiang Yang, Shenxi Chen

**Affiliations:** ^1^ Hubei Key Laboratory of Quality and Safety of Traditional Chinese Medicine and Health Food, Jing Brand Co., Ltd., Daye, China; ^2^ Guangxi Tianlongquan Wine Industry Co., Ltd., Luocheng, China

**Keywords:** rice-flavor baijiu, semisolid state fermentation, microbial diversity, metagenomic sequencing, flavors

## Abstract

This study aimed to explore the microbial contribution to flavor compound production by analysing the succession patterns and metabolic functional characteristics of microbial communities during *Jiuqu* preparation, saccharification, and fermentation processes of rice-flavor Baijiu (RFB). The physicochemical parameters during RFB fermentation were systematically monitored, and the volatile flavor profile was characterized using headspace solid-phase microextraction gas chromatography‒mass spectrometry (HS-SPME–GC–MS). Concurrently, metagenomic sequencing was employed to elucidate the microbial community structure and its temporal dynamics throughout the fermentation process. The results of the physicochemical parameters revealed that the reducing sugar content peaked at the end of saccharification and subsequently decreased throughout fermentation, whereas the total acid and total ester contents progressively increased, reaching maximum levels at the fermentation endpoint and maintaining stability. HS-SPME–GC–MS analysis revealed 84 volatile flavor compounds including phenylethanol, ethanol, dimethyl ether, isopentyl alcohol, and acetic acid. Notably, compounds such as L-ethyl lactate, diethyl succinate, and isobutanol were initially synthesized during saccharification and subsequently accumulated during fermentation, emerging as major flavor constituents. Ascomycota and Mucoromycota dominated the fungal community (average relative abundance >1%), whereas Firmicutes and Proteobacteria prevailed among the bacterial phyla. Six genera, *Lichtheimia*, *Kluyveromyces*, *Lacticaseibacillus*, *Lactobacillus*, *Limosilactobacillus*, and *Schleiferilactobacillus* were identified as primary contributors to flavor production during fermentation. Functional analysis revealed that microbial metabolism in fermented mash primarily involved amino acid and carbohydrate metabolism, with glycoside hydrolases (GHs) and glycosyl transferases (GTs) serving as key carbohydrate-active enzymes. This study could improve the comprehensive understanding of the brewing mechanism of RFB and provide a theoretical basis for the development and utilization of microbial resources in the fermented grains and the improvement of RFB quality.

## 1 Introduction

**FIGURE 1:**
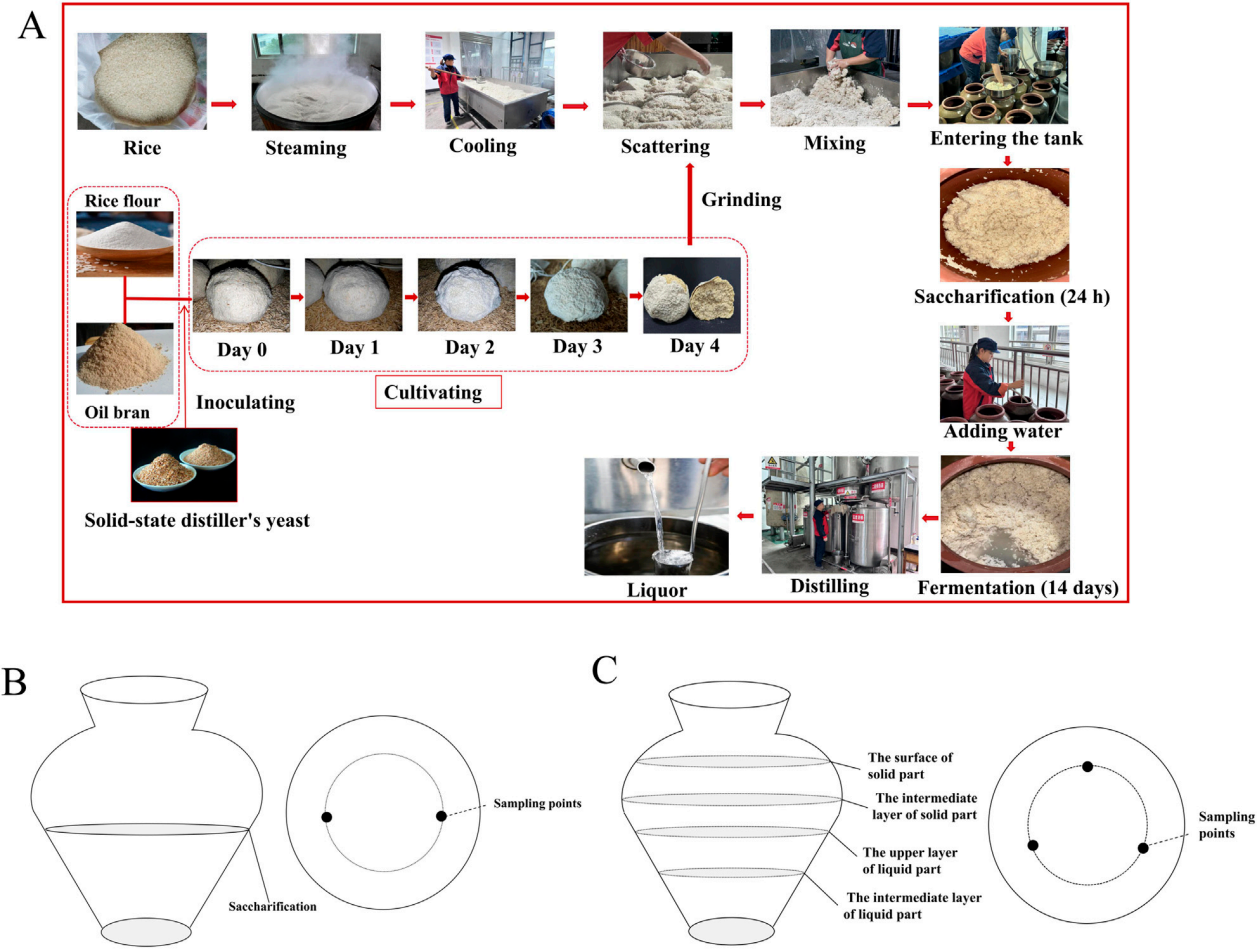
Production process of rice-flavor Baijiu **(A)** and sampling locations of saccharification **(B)** and fermentation **(C)**.

As one of the world’s oldest distilled spirits, Chinese Baijiu represents a significant cultural heritage, among which rice-flavor Baijiu (RFB) has emerged as a distinctive variety with profound historical roots and is particularly popular in southern China because of its unique flavor proSankuanfile (Wu et al., 2024). The production of RFB primarily involves four key stages: *Jiuqu* preparation, saccharification, fermentation, and distillation. *Jiuqu*, a fermentation starter, serves as a vital source of diverse enzymes and microorganisms that drive the essential biochemical reactions throughout the brewing process (Zheng et al., 2011). Rice flour and oil bran are used for RFB *Jiuqu* production as raw materials, and they are mixed and kneaded into a Mantou shape. Then, they are transferred into the *Jiuqu* incubation room and incubated at 30 °C for 48 h. After that, *Jiuqu* is obtained after drying naturally for 2 days (Figure 1A). The RFB fermentation process employs a unique combination of solid-state saccharification and semisolid fermentation, typically utilizing rice as the primary raw material. Notably, water is added at the end of the saccharification stage. The fermentation cycle is relatively short, lasting from 8–14 days. Owing to this distinctive brewing method, RFB has significant differences in flavor profile and production characteristics corresponding to the three major Baijiu styles in China: Maotai-flavor, Light-flavor, and Luzhou-flavor (Wang et al., 2023). Compared with these three Baijiu varieties, which have spicy tastes, the main sensory characteristics of RFB are “elegant honey flavor, soft mouth, clean mouth and pleasant taste” (Hu et al., 2021). The distinctive olfactory profile is attributed to the synergistic contribution of three volatile organic compounds: ethyl lactate, ethyl acetate, and β-phenylethanol (Liang et al., 2024).

Flavor compounds are the core factors determining the quality of rice-flavor Baijiu, whereas microorganisms, as the “invisible engineers” of the fermentation process, participate in key biochemical processes such as sugar conversion, ester synthesis, and organic acid accumulation through metabolic activities, directly influencing the formation and regulation of the liquor’s flavor profile. During the brewing of RFB, the microbial community mainly comprises *Saccharomyces*, *Rhizopus, Lactobacillus, Pediococcus* and *Weissella* (Liu, et al., 2018; Hu et al., 2021). *Rhizopus* can degrade large molecules (e.g., starch, lipids, and protein) into simpler compounds (e.g., glucose and fructos) by secreting extracellular enzymes, such as hydrolytic enzymes, proteases, and lipases. These enzymatic activities not only increase nutrient availability but also promote the proliferation of other essential microorganisms (Kim et al., 2011; Wu et al., 2021). Yeast is an important microorganism that produces ethanol and flavor compounds; for example, the major esters in RFB, such as ethyl acetate, and key alcohols, such as β-phenylethanol, are all produced by yeast (e.g., *Saccharomycopsis,* and *Saccharomyces*) metabolism (Maurizio, et al., 2016). Lactic acid bacteria are the absolute dominant bacteria in RFB, and they can produce organic acids and promote the formation of ester substances (Zhang et al., 2022). Many microbial communities are present during Baijiu fermentation, but only the core microbes play major roles in promoting flavor production. They are not only responsible for generating a variety of flavor components, but also maintain the ecological balance between microorganisms, and affect the quality of Baijiu (Song et al., 2017). In recent years, several scholars have researched the flavor and microbial community of RFB. Wang et al. revealed the mechanism of how microbial community structure affects the production of higher alcohols in RFB (Wang et al., 2023). Rao and colleagues explored the influence of different raw materials on the flavor of RFB (Rao et al., 2023). Hu et al. compared the differences in microbial community structure among three basic flavor types of Baijiu: light-flavor, rice-flavor, and Nongxiang flavor (Hu et al., 2021). Although these studies have provided valuable insights into the flavor and microbial community structure of RFB, the mechanism of flavor formation in its traditional brewing process remains unclear, and the complex correlations between microbial communities and key flavor compounds need to be further elucidated. Therefore, analysing the dynamics of the microbial community structure, the patterns of flavor compound changes, and their intrinsic relationships in the brewing process of RFB is of great theoretical and practical importance for revealing its flavor formation mechanism, optimizing brewing techniques, and enhancing product quality.

With the development of omics technology, metagenomics has become an excellent tool for application in the current research field of food microbiology. This technology directly extracts DNA from all microbial communities in specific environments and uses gene screening and sequencing to analyse their structure and functional genes. This method not only avoids the complex steps of microbial screening and cultivation, reducing time costs, but can also detect low-abundance microorganisms, improving detection efficiency and accuracy. In recent years, metagenomic sequencing has been widely applied in the field of fermented foods, such as dough (Liu et al., 2024), tea (Li et al., 2018), liquor (Xue et al., 2023), wine (Gao et al., 2024; Zambianchi et al., 2023), and vinegar (Li et al., 2023), and has yielded a series of research results. Therefore, in this study, we utilized metagenomic sequencing technology to analyse the structural changes in the microbial community during the brewing process of RFB, and identified core functional microbial groups. Simultaneously, we combined headspace solid-phase microextraction–gas chromatography-mass spectrometry (HS-SPME–GC–MS) to qualitatively analyse the flavor compounds present during the brewing process. Finally, through multivariate statistical analysis, we constructed a correlation network between the microbial community and flavor compounds. This study provides a scientific basis for a deeper understanding of the microbial metabolic mechanism underlying the flavor formation of RFB, and offers theoretical support for the modernization and quality improvement of traditional Baijiu brewing techniques.

## 2 Materials and methods

### 2.1 Sample collection

The *Jiuqu* and fermentation mash samples were collected from Tianlongquan Liquor Industry Co., Ltd. Samples were collected from the *Jiuqu*, early, middle, and late stages of fermentation, i.e., at 0, 3, 5, 9, and 14 days, and they were labelled as JQ, THJS, FJ3D, FJ5D, FJ9D,and FJ14D, respectively. Each sampling time point included three biological replicates collected from three distinct pottery jars, with a total of four batches sampled for comprehensive analysis. The sampling method referred to a previous study with some modifications (Wang et al., 2024). As shown in Figure 1B, during the collection of the finished saccharification mash sample, the sampling positions were fixed at two points within the middle layer of the mash. Approximately 100 g of sample was collected from each point, and the six samples (from three jars) were thoroughly mixed. The fermentation stage of RFB exhibited a biphasic conditions, comprising both solid and liquid phases, with the solid phase remaining buoyant throughout the process. To ensure sampling uniformity, the solid and liquid phases were sampled separately and then combined. As shown in Figure 1C 0 g of fermented mash was collected from three fixed points at the interface of the solid and liquid layers in each pottery jar. All of these samples were mixed thoroughly, and 50 g of the homogenized mixture was frozen at −80°C for metagenomic sequencing. The remainder was stored at −4°C for further analysis of physicochemical properties and flavor components.

### 2.2 Methods

#### 2.2.1 Physicochemical factors determination

The acidity was determined as described by Lin et al. (2022). The specific method was as follows: 1 mL of the sample filter solution was placed in a 150 mL conical flask, followed by the addition of 50 mL of ultrapure water and 2 drops of phenolphthalein indicator. The solution was then titrated with 0.1 mol/L NaOH standard solution until a faint red color appeared and persisted for 30 s, marking the endpoint. The reducing sugar content of the samples was measured via Feiling reagent method (AL-Khafaji., 2024). The determination of total esters content was performed using an Agilent 7890B gas chromatograph equipped with a flame ionization detector (GC-FID) and a DB-FFAP column (Agilent Technologies, California, United States). The analytical procedure was conducted as follows: 50 mL of mash was centrifuged at 8,000 r/min for 10 min. Subsequently, 1 mL of supernatant was precisely pipetted and mixed with 1 mL of anhydrous ethanol. The mixture was filtered through a 0.22 µm membrane filter, followed by the addition of 10 µL of mixed internal standard solution (containing 2-ethylhexanol, tert-amyl alcohol, and amyl acetate) to 1 mL of the filtrate. After thorough homogenization, the prepared sample was injected for analysis. Chromatographic conditions were optimized as: The injector operated in split mode with a split ratio of 30:1 at 25°C. The oven temperature program initiated at 35°C (held for 1 min), followed by a temperature ramp to 120°C at 3.5°C/min, then increased to 190°C at 20°C/min. High-purity nitrogen (99.999%) served as the carrier gas with a constant flow rate of 1 mL/min. All physicochemical factors of the *Jiuqu* samples and the fermented mash samples were measured in triplicate.

#### 2.2.2 Determination of volatile compounds

The analysis of volatile components in *Jiuqu* and fermented mash was performed using an Agilent 7890B gas chromatograph coupled with a 5977B mass selective detector (HS-SPME–GC–MS) (Agilent Technologies, California, United States). Specifically, 2 g of *Jiuqu* sample was weighed and transferred into a 20 mL headspace vial containing 5 mL of saturated sodium chloride solution and 20 µL of an internal standard solution (2-octanol and 2-ethyl hexanol). For the fermented mash sample, the mixture was centrifuged at 8,000 rpm for 5 min. The supernatant (8 mL) saturated with NaCl was spiked with an internal standard solution (20 µL) of 2-octanol (162.68 mg/L) and 2-ethyl hexanol (197.90 mg/L) in a 20 mL headspace injection vial. The headspace vial was placed in the automatic microextraction device and extracted at 50 °C for 45 min with SPME fibres of 50/30 μm DVB/CAR/PDMS (Supelco Co., PA, United States). The GC‒MS running conditions were as follows: the oven temperature program was initiated at 50°C, increased to 220 °C at a rate of 3.0 °C/min, and then held for 5 min. Helium was used as the carrier gas at a constant flow rate of 1.0 mL/min. Electron ionization (EI) mass spectrometry mode was employed with an ionization energy of 70 eV. The ion source temperature was 230°C, and full-scan mode was applied across the mass range of 20–500 amu (Sun et al., 2022). Volatile compounds were identified by matching with the National Institute of Standards and Technology (NIST 20) library (Gaithersburg, MD, United States), and the matching masses were more than 80%. The relative concentration of volatile compounds was calculated based on the ratios of the volatiles’ peak areas to the internal standards’ peak areas.

#### 2.2.3 DNA extracting and metagenomic sequencing

Total microbial genomic DNA samples were extracted using the OMEGA Mag-Bind Soil DNA Kit (M5635-02) (Omega Bio-Tek, Norcross, GA, United States). The quantity and quality of extracted DNA were measured using a Qubit™ 4 Fluorometer and agarose gel electrophoresis, respectively. The extracted microbial DNA was processed to construct metagenome shotgun sequencing libraries with insert sizes of ∼400 bp by using Illumina TruSeq Nano DNA LT Library Preparation Kit (Illumina, United States). Each library was sequenced by Illumina NovaSeq platform (Illumina, United States) with PE150 strategy at Personal Biotechnology Co., Ltd. (Shanghai, China).

Raw sequencing reads were processed to obtain quality-filtered reads for further analysis. Firstly, sequencing adapters were removed from sequencing reads using Cutadapt (v1.2.1) (Martin, 2011). Secondly, low quality reads were trimmed using a sliding-window algorithm in fastp (v0.23.2) (Chen et al., 2018). Thirdly, reads were aligned to the host genome of fungal and bacterial using Minimap2 (v2.24-r1122) to remove host contamination (Li, 2018). Megahit (v1.1.2) was used for assembly of reads in each sample using the meta-large presetted parameters (Li et al., 2015). The generated contigs (longer than 300bp) were then pooled together and clustered using mmseqs2 with “easy-linclust” mode, setting sequence identity threshold to 0.95 and covered residuse of the shorter contig to 90% (Steinegger and SöDing, 2017). Prodigal (V2.6.3) was used to predict the genes in the contigs (Hyatt et al., 2010). CDS sequences of all samples were clustered by mmseqs2 with “easy-cluster” mode, setting protein sequence identity threshold to 0.95 and covered residue of the shorter contig to 90%. To assess the abundances of these genes, the high-quality reads from each sample were mapped onto the predicted gene sequences using Minimap2 with “-ax sr--sam-hit-only” and using featureCounts to count the number of reads aligned to gene sequences, i.e., the Reads Count (RC) for each gene (Liao et al., 2014).

The functionality of the non-redundant genes were obtained by annotated using mmseqs2 with the “search” mode against the protein databases of KEGG, EggNOG and CAZy databases and using Diamond (v2.0.15) against CARD, Metacyc, MCycDB, PCycDB, NCycDB and SCycDB database, respectively.

### 2.3 Statistical analyses

Here, all experiments were repeated three times and the results expressed as mean ± standard deviation. Based on the taxonomic and functional profiles of non-redundant genes, LEfSe (Linear discriminant analysis effect size) was performed to detect differentially abundant taxa and functions across groups using the default parameters. Beta diversity analysis was performed to investigate the compositional and functional variation of microbial communities across samples using Bray-Curtis distance metrics and visualized via principal coordinate analysis (PCoA). The spearman correlation coefficient analysis between the top 20 bacterial and fungal genera in terms of relative abundance and the main flavor compounds was performed using the OmicStudio tools at https://www.omicstudio.cn/tool. And the *r* > 0.5 and *p* < 0.05 were used as thresholds to find the visualization object, then used Cytoscape 3.9.1 to visualize the interaction relationship between microorganisms and flavor compounds, characterizing the contribution of microorganisms to flavor compounds. OriginPro (Version 2021. OriginLab Corporation, Northampton, MA, United States) was used for the rest of the graphics.

## 3 Results

### 3.1 Changes in physicochemical indices

**FIGURE 2:**
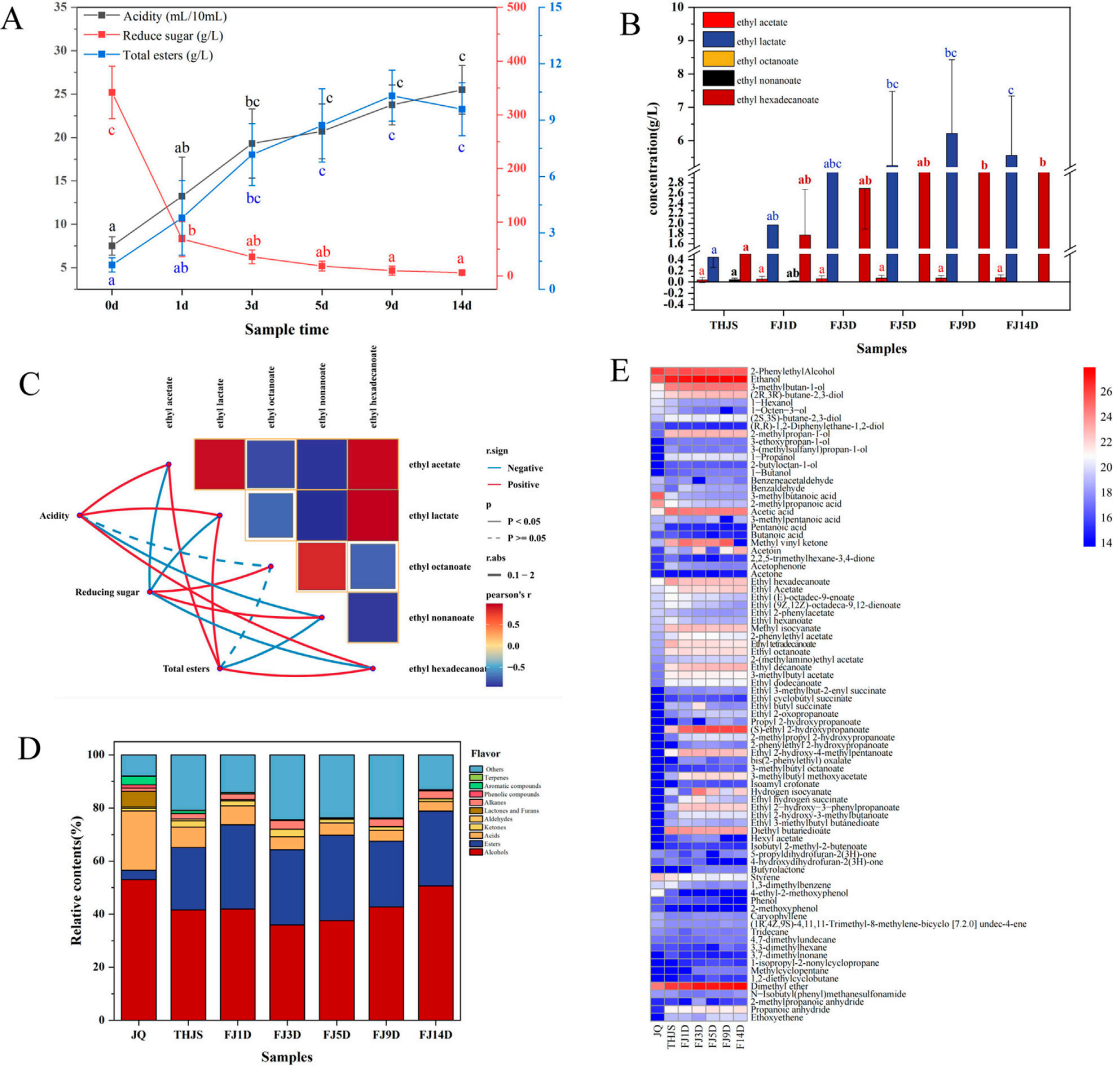
Changes of physicochemical factors and flavor substances during the fermentation of RFB. **(A)** The total concentration of each physicochemical index. **(B)** The concentration of each ester. Note: For two groups of markers with significant differences (*p* < 0.05), and the same letter indicates no significant difference; different letters indicate significant differences. **(C)** Correlation analysis between physicochemical indicators and major ester compounds. The color of the connecting lines indicates correlation: red represents a positive correlation, and blue represents a negative correlation, and the thickness of the lines indicates the strength of the correlation, while the solid and dashed lines indicate significant differences. **(D)** Proportion of flavor components in RFB *Jiuqu* and fermented mash. **(E)** Heatmap showing the frequency distribution of volatile components in *Jiuqu* and fermented mash of RFB.

Here, the acidity, reducing sugars, and total esters were determined in all the samples. As shown in the Figure 2A, the acidity of the fermented mash tended to increase throughout the entire fermentation process, and the acidity reached a high value on the 14th day. The acidity value was three times greater than that at the end of the saccharification period. Moreover, the acidity growth trend in the early stage of fermentation (d0–d3) was faster than that in the late stage of fermentation (d5–d14). Before the fermentation stage, saccharification broke down a large amount of starch in the fermented mash into reducing sugars and peaked, with a reducing sugar content of 341.7 g/L. The saccharification rate then decreased throughout fermentation, and the reducing sugar content of the fermented mash tended to decrease. In addition, the content of reducing sugars significantly decreased in the early stage of fermentation (d0–d3) and tended to stabilize in the late stage of fermentation (d5–d14). The total ester content refers to the total contents of ethyl acetate, ethyl butanoate, isoamyl acetate, ethyl hexanoate, ethyl lactate, ethyl octanoate, ethyl nonanoate and ethyl hexadecanoate. As shown in Figure 2B, the total ester content gradually increased from saccharification to fermentation, reached its maximum value (10.3 g/L) on the 9th day of fermentation, and then stabilized. The contents of ethyl lactate and ethyl hexadecanoate were greater than those of the other esters detected. In addition, the contents of ethyl acetate, ethyl lactate, and ethyl hexadecanoate increased with fermentation, whereas the content of ethyl octanoate decreased. Furthermore, ethyl nonanoate was only produced at the end of saccharification and on the first day of fermentation, whereas ethyl butanoate, isoamyl acetate and ethyl hexanoate were not detected in the fermented mash. To further reveal the impact of physicochemical indicators on the formation of flavor compounds, we analysed the correlations between physicochemical factors and key ester compounds in fermented mash (Figure 2C). Acidity and the total ester contents were positively correlated with ethyl acetate, ethyl lactate, and ethyl hexadecanoate, whereas reducing sugars were positively correlated with ethyl octanoate and ethyl nonanoate.

### 3.2 Analysis of flavor compounds during fermentation process

Volatile flavor compounds play a crucial roles in determining the flavor and quality of Baijiu. In this study, the HS-SPME-GC-MS method was employed to identify the primary volatile flavor substances in RFB *Jiuqu* and fermented mash. A total of 84 volatile flavor compounds were detected, categorized as follows: 35 esters, 5 ketones, 6 acids, 2 aldehydes, 14 alcohols, 3 lactones, 2 aromatics, 3 phenols, 1 terpene, 8 alkanes, and 5 other volatile compounds. Alcohols emerged as the most abundant flavor compounds (Figure 2D), constituting 52.97%, 41.47%, 41.96%, 35.92%, 37.55%, 42.68%, and 50.57% of the flavor compounds in the JQ, THJS, FJ1D, FJ3D, FJ5D, FJ9D, and FJ14D samples, respectively. In the JQ group, 2-phenylethanol and ethanol were the predominant alcohols. However, 3-methylbutan-1-ol, 2-methylpropan-1-ol, and (R,R)-2,3-butanediol became the major alcohols during the saccharification and fermentation stages. Notably, the concentration of 2-phenylethanol decreased as fermentation progressed, whereas the levels of 3-methylbutan-1-ol, 2-methylpropan-1-ol, and (R,R)-2,3-butanediol increased. The relative contents of major esters in the JQ, THJS, FJ1D, FJ3D, FJ5D, FJ9D, and FJ14D samples from RFB were significantly lower than those in the alcohols samples (Figure 4A), accounting for 3.58%, 23.54%, 31.78%, 28.39%, 32.16%, 24.82%, and 28.24%, respectively. These results suggested that ester compounds began to accumulate and increase during the saccharification and fermentation stages. Among these esters, ethyl (S)-2-hydroxypropanoate, a key flavor component of RFB, presented the highest relative content, which increased gradually as fermentation advanced. This observation aligned with our physicochemical property analysis. Additionally, other esters including diethyl butanedioate, ethyl 2-hydroxy-4-methylpentanoate, ethyl acetate, ethyl hexadecanoate, ethyl decanoate, ethyl octanoate, and ethyl tetradecanoate (Figure 2E), had high relative contents during the saccharification and fermentation stages. The relative content of acids in the JQ sample was 22.39%, which was significantly greater than those in the saccharification and fermentation samples (<10%). In the JQ sample, the predominant acids were 3-methylbutanoic acid and 2-methylpropanoic acid. However, during the saccharification and fermentation stages, acetic acid became the most abundant acid. Additionally, dimethyl ether was present in substantial quantities in RFB, and was detectable in both the JQ sample and throughout the fermentation mash. Its content increased gradually as fermentation progressed. These findings highlighted the significant influence of these compounds on the quality and distinctive style of RFB.

### 3.3 Microbial community composition during the brewing process of RFB

**FIGURE 3:**
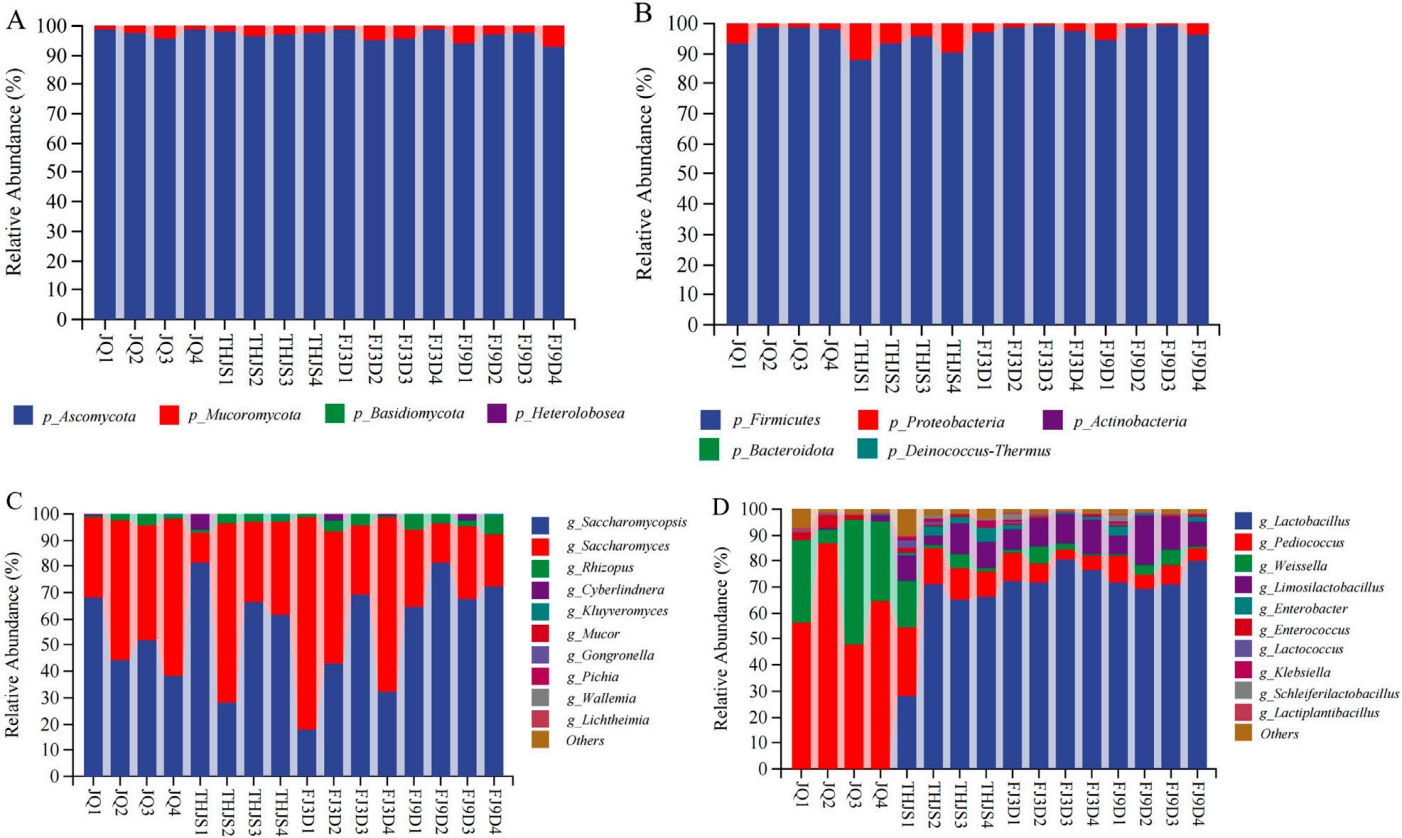
The composition of microbial community in fermented mash in the production of rice-flavor Baijiu. Fungi at phylum **(A)**, and genus **(C)** levels. Bacteria at phylum **(B)**, and genus **(D)** levels. The rank in genera (top 10) was obtained according to the average relative abundance of all samples.

Here, the representative RFB *Jiuqu* and fermented mash at four different stages (JQ, THJS, FJ3D, FJ9D) of the brewing process were selected for metagenomic sequencing. To understand the characteristics of microbial communities in different fermentation stages of RFB, the composition and diversity of the microbial community were analysed. At the phylum level (Figures 3A,B), fungi included Ascomycota and Mucoromycota (average relative abundance>1%). Ascomycota was identified as the absolute dominant fungal phylum, with relative abundances of 97.8%, 97.4%, 97.2%, and 95.3% at the four time points. The bacterial communities consisted primarily of Firmicutes and Proteobacteria (average relative abundance>1%). Firmicutes was the absolute dominant bacterial phylum, with the relative contents of 97.4%, 91.9%, 98.1%, and 97.3% at the four time points, respectively.

The top 10 fungal and bacterial genera, which were determined based on the average relative abundance of all the samples, are shown in Figures 3C,D. *Saccharomycopsis*, *Saccharomyces,* and *Rhizopus* were the three dominant genera, with average relative abundances >1%. In *Jiuqu* and during the saccharification stage, *Saccharomycopsis*, *Saccharomyces,* and *Rhizopus* had average relative abundances of 50.7%–59.4%, 36.4%–46.8%, and 2.1%–2.5%, respectively. As fermentation progressed, the average relative abundance of *Saccharomycopsis* decreased rapidly, whereas that of *Saccharomyces* increased sharply. In the early stage of fermentation (Day 3), the average relative abundance of *Saccharomyces* increased to 55.9%, whereas that of *Saccharomycopsis* decreased to 40.6%. At this time, *Saccharomyces cerevisiae* rapidly proliferated to produce alcohol, becoming the absolute dominant fungal species during this period. However, in the late stage of fermentation (Day 9), *Saccharomyces* was replaced by *Saccharomycopsis*, and *Saccharomycopsis* became the dominant fungal genus again, with an average relative abundance of 71.6%, whereas *Saccharomyces* decreased to 23%. *Lactobacillus*, *Pediococcus*, *Weissella*, and *Limosilactobacillus* were the four dominant bacterial genera with average relative abundances greater than 1%. In the *Jiuqu* samples, the dominant bacterial genera were *Pediococcus* (63.8%), *Weissella* (28.9%), and *Enterococcus* (2.37%). During the saccharification stage, the dominant bacterial genera were *Lactobacillus* (57.6%), *Pediococcus* (15.7%), *Limosilactobacillus* (8.8%), *Weissella* (6.2%), *Enterobacter* (3.3%), *Lactococcus* (1.3%), and *Klebsiella* (1.3%). In the early stage of fermentation (Day 3) and the late stage of fermentation (Day 9), the dominant bacterial genera were *Lactobacillus*, *Limosilactobacillus*, *Pediococcus*, *Weissella,* and *Enterobacter*.

**FIGURE 4:**
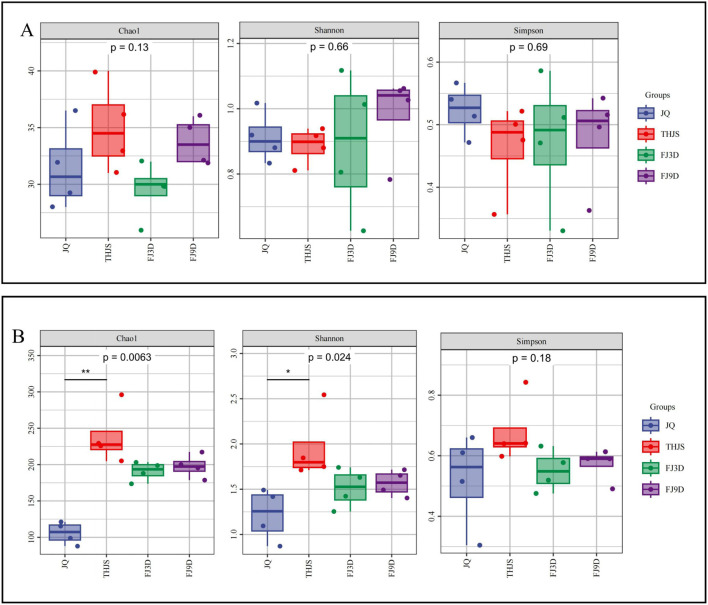
Alpha diversity of fungi **(A)** and bacteria **(B)** based on metagenomic sequencing. Note: For two groups of markers with significant differences (0.01 < *p* ≤ 0.05 marked as *, 0.001 < *p* ≤ 0.01 marked as * *, *p* ≤ 0.001 marked as ***).

The Chao1, Shannon and Simpson indices of the *Jiuqu* and fermented mash samples were determined with intergroup difference tests (Dunn’s test *post hoc* test) (Figure 4). The bacterial diversity index was significantly greater than that of fungi (*p* < 0.05) during saccharification and fermentation. The lack of significant differences in the fungal Chao1, Simpson, and Shannon indices indicated that the fungal richness and diversity were similar between the *Jiuqu* and fermented mash samples. The bacterial diversity index results revealed that the Chao1, Simpson, and Shannon indices of the THJS sample were 238.94, 0.68, and 1.96, respectively, which were the highest among the four groups of samples. The Chao1 index of the THJS sample was significantly different from that of the JQ sample (*p* < 0.01), and the Shannon index was different from that of the JQ sample (*p* < 0.05), indicating that the bacterial richness and diversity were the highest in the THJS sample, showing a trend of first increasing and then decreasing throughout the entire brewing process.

**FIGURE 5:**
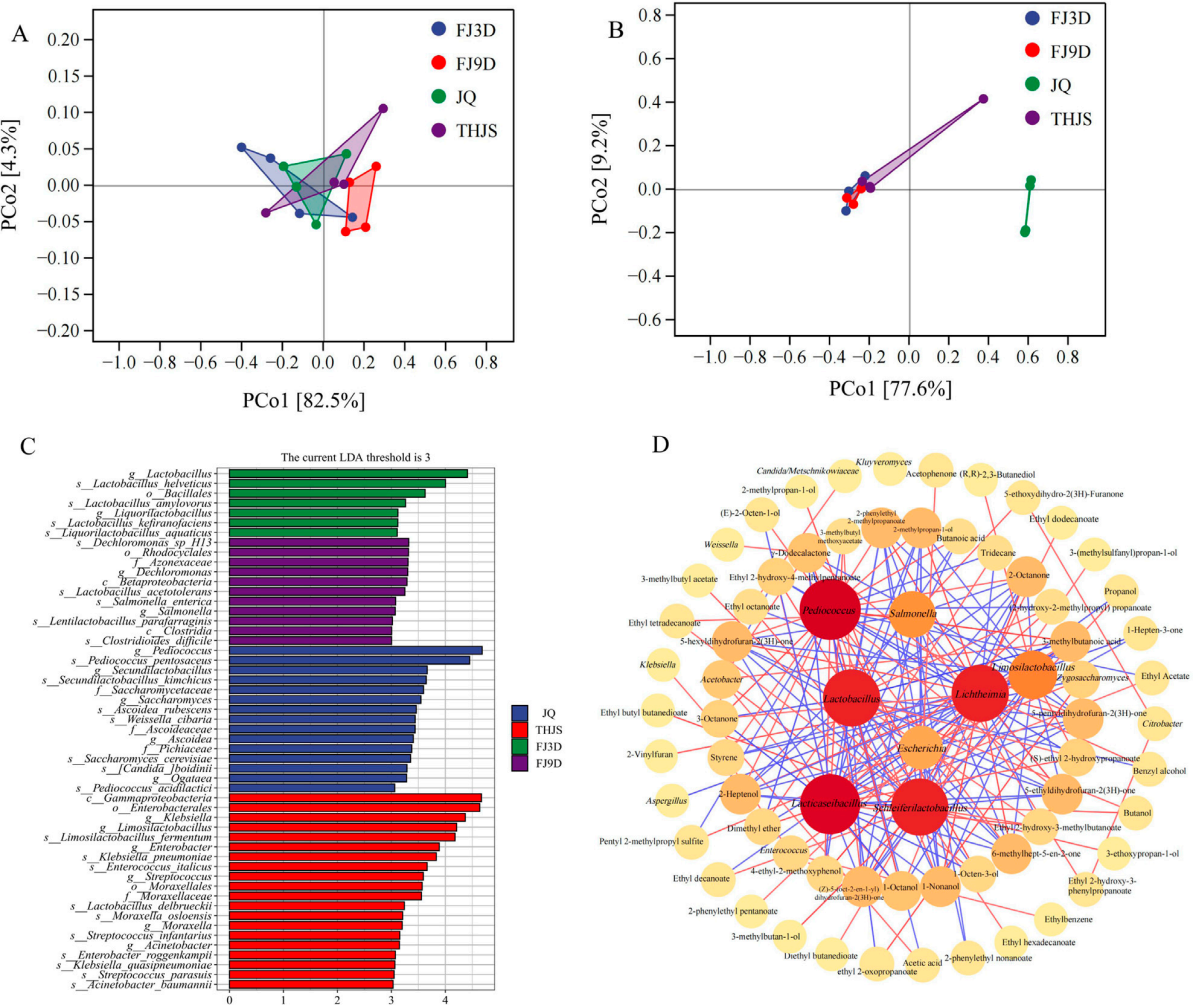
Principal Coordinates Analysis (PCoA) of fungi **(A)** and bacteria **(B)**. **(C)** LEfSe analysis of microbial species in samples of RFB at different brewing stages (LDA >3). **(D)** Correlation network between main microbial genera and volatile compounds. Significant spearman’s correlations (|*r*| > 0.5, *p* < 0.05) were shown in diagram. Microbes and volatile substances were represented by red, orange and yellow modules, and the darker the modules color, the stronger the correlation. And the red and blue lines refer to the positive and negative correlations between dominant genera and aroma compounds, respectively.

The PCoA plot (Figures 5A,B) demonstrated that the nodes representing samples in the four different stages of the fungal community brewing process were relatively close, indicating that there was a high similarity in fungal biodiversity in the *Jiuqu*, saccharification and fermentation stages. The sample nodes representing the bacterial community in the JQ sample were clearly separated from those in the THJS, FJ3D, and FJ9D samples, whereas the replicate sample points in the THJS, FJ3D, and FJ9D clustered together, indicating significant differences in the composition of the bacterial community between *Jiuqu* and the saccharification and fermentation stages. Furthermore, as the fermentation process progressed, the bacterial community structure in the saccharified mash gradually stabilized.

To identify characteristic microorganisms across different groups, Linear Discriminant Analysis (LDA) Effect Size (LEfSe) was conducted. As shown in Figures 5C microbial strains were significantly enriched in JQ, 20 strains in THJS, 7 strains in FJ3D samples, and 11 strains in FJ9D samples. In the JQ, the biomarkers included *Pediococcus pentosaceus*, *Pediococcus acidilactici*, *Secundilactobacillus kimchicus*, *Saccharomyces cerevisiae*, *Ascoidea rubescens*, and *Weissella cibaria*. In contrast, *Klebsiella pneumoniae*, *Limosilactobacillus fermentum*, *Enterococcus italicus*, *Streptococcus infantarius*, *Streptococcus parasuis*, and *Acinetobacter baumannii* were identified as biomarkers in THJS. In the FJ3D samples, the dominant characteristic microorganisms were *Lactobacillus helveticus*, *Lactobacillus amylovorus*, *Lactobacillus kefiranofaciens*, and *Lactobacillus aquaticus*. By the end of fermentation (FJ9D), the biomarkers shifted to *Dechloromonas* sp. H13, *Salmonella enterica*, and *Lactobacillus acetotolerans*.

### 3.4 Correlation analysis between microorganisms and flavor compounds

The relationships between major microbial genera and volatile components were visualized using Cytoscape. Correlation networks further confirmed that these microorganisms contributed greatly to the accumulation of flavor components (Figure 5D). *Lichtheimia* was the most connected fungal genus, whereas *Pediococcus*, *Lacticaseibacillus*, *Lactobacillus*, and *Schleiferilactobacillus* were the four most connected bacterial genera. Among the fungal genera, *Mucor* and *Rhizopus* were positively related to ethanol, and *Aspergillus*, *Zygosaccharomyces,* and *Pichia* were positively related to esters and acids such as ethyl acetate and ethyl (S)-2-hydroxypropanoate and acetic acid. *Lichtheimia* was positively related to 2-phenylethyl alcohol and acids such as 3-methylbutanoic acid and 2-methylpropanoic acid, whereas *Lichtheimia* and *Kluyveromyces* were negatively correlated with alcohols, acetic acid, and esters. The bacterial genera *Lacticaseibacillus*, *Lactobacillus*, *Schleiferilactobacillus,* and *Limosilactobacillus* were positively correlated with alcohols, esters, and acids such as acetic acid, but they were negatively correlated with acids such as 3-methylbutanoic acid and 2-methylpropanoic acid. The genus *Pediococcus* was positively correlated with the main acidic substance 3-methylbutanoic acid and the main alcohol substance 2-phenylethyl alcohol and negatively correlated with the main alcohol substance (R,R)-2,3-butanediol and the main ester substances ethyl acetate and ethyl (S)-2-hydroxypropanoate, among others.

### 3.5 Metabolism and pathways predicted by metagenomic sequencing

**FIGURE 6:**
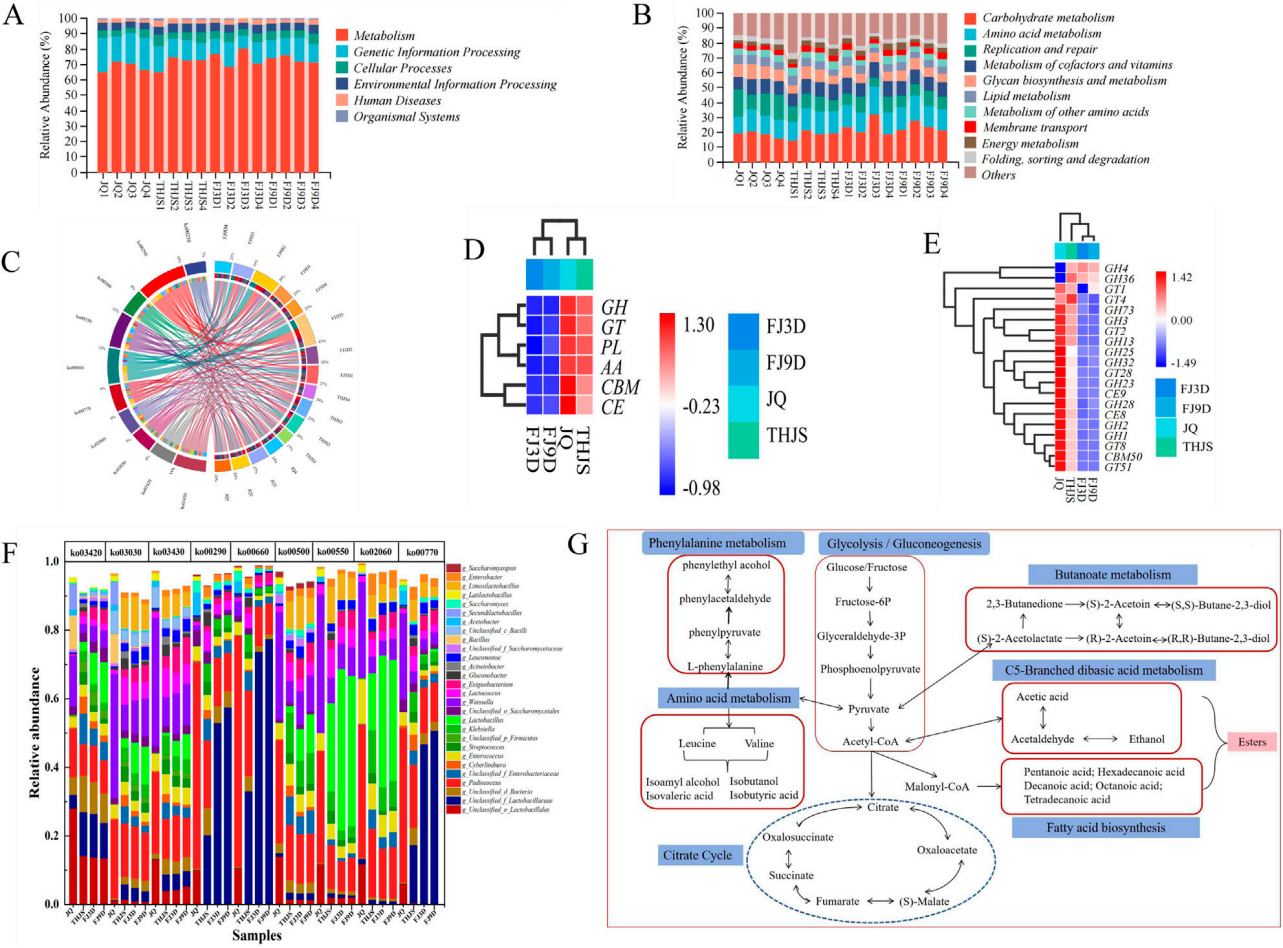
Functional classification of microorganisms in KEGG and CAZy functional database and metabolic pathway of key flavor compounds of RFB. **(A)** Level 1 in KEGG, **(B)** Level 2 in KEGG, **(C)** Level 3 in KEGG, **(D)** Level 1 in CAZy, **(E)** Level 2 in CAZy, **(F)** Microbial contribution to KEGG level 3 pathways, **(G)** Predicted metabolic pathway.

Microorganisms cultivated at different brewing stages presented distinct microbial metabolic activities. To clarify the metabolic differences between groups, functional annotation was performed using the CAZy and KEGG databases. The annotation results of the functional genes based on the KEGG database are shown in Figure 6A. All the genes were classified into six major categories at KEGG level 1, including metabolism, genetic information processing, cellular processes, environmental information processing, human diseases, and organismal systems. Metabolism was the most annotated functional gene, and its content increased from *Jiuqu* to the fermentation stage, reaching the highest abundance of 74.08% on the third day of fermentation. In the functional abundance map under KEGG level 2 classification, 11 pathways fell under the metabolism category (Figure 6B). The carbohydrate metabolism pathway had the greatest relative abundance, followed by pathways involved in amino acid metabolism and replication and repair. Replication and repair were the main pathways involved in *Jiuqu*, whereas amino acid metabolism was the main pathway involved in saccharification and fermentation. In the metabolic pathway analysis under the KEGG level 3 classification (Figure 6C), the microbial metabolic pathways in *Jiuqu* were relatively abundant, with the main metabolic pathways being replication and repair (ko03030, ko03420, ko03430). During the saccharification stage, the main metabolic pathways gradually became those involving amino acid metabolism, namely, the biosynthesis of valine, leucine, and isoleucine (ko00290), and during the fermentation stage, the main metabolic pathway became those involving carbohydrate metabolism, namely, C5 branched dibasic acid metabolism (ko00660) and starch and sucrose metabolism (ko00500). KEGG analysis revealed that the microbial community primarily proliferated during the early stage of fermentation. As fermentation progressed, carbohydrates, amino acids, and other substances in the microbial community raw materials were metabolized to generate small-molecule compounds (such as ethanol, lactic acid, acetic acid, and ethyl ester).

To better understand the functional potential of the brewing microorganisms, the distribution of pathways in the KEGG database was determined. As shown in Figure 6F, the main contributors to various metabolic pathways were bacteria, especially lactic acid bacteria, such as *Pediococcus* and *Weissella*. The genera *Unclassified_f_Lactobacillaceae*, *Pediococcus*, *Weissella*, *Unclassified_o_Lactobacillales,* and *Limosilactobacillus* were the top 5 closely related to carbohydrate and amino acid metabolism. Among these genera, *Pediococcus* had a relatively high abundance in *Jiuqu*. During the saccharification stage, *Unclassified_f_Lactobacillaceae* rapidly proliferated and became the bacterial genus that contributed the most to ko00290 (valine, leucine, and isoleucine biosynthesis) and ko00660 (C5-branched dibasic acid metabolism) by replacing *Pediococcus* during the fermentation stages. The ko00500 metabolic pathway (starch and sucrose metabolism) is important in the brewing process. *Pediococcus* and *Weissella* mainly exerted metabolic functions related to ko00500 in *Jiuqu*, and after entering the saccharification and fermentation stages, *Klebsiella*, *Lactobacillus*, *Unclassified_f_Enterobacteriaceae*, *Limosilactobacillus*, *Cyberlindera*, *Streptococcus*, *Exiguobacterium,* and *Enterobacter* also began to participate in starch and sucrose metabolism.

The CAZy database is a professional database for studying carbohydrate enzymes, and the annotation results of functional genes derived from the database are shown in Figures 6D,E. All genes were classified into six major categories at level 1 of CAZy. The numbers of annotations for glycoside hydrolases (GHs), glycosyl transferases (GTs), polysaccharide lyases (PLs), auxiliary activities (AAs), carbohydrate esterases (CEs), and carbohydrate-binding modules (CBMs) in *Jiuqu* were relatively high. When the saccharification stage occurred, the numbers of annotations for glycoside hydrolase (GH), glycosyltransferase (GT), polysaccharide lyase (PL) and auxiliary oxidoreductase (AA) were particularly high. At CAZy level 2, the functional abundance clustering results revealed that the glycoside hydrolases in *Jiuqu* were predominantly classified into lactase (GH1), β-mannosidase (GH2), (1,4)-α-D-glucan 1-α-D-glucosidase (GH13), lysozyme (GH25), polygalacturonase (GH28), and fructosyltransferase (GH32). These results indicated that microbial activity in *Jiuqu* was highly dynamic, involving extensive carbohydrate decomposition and the breakdown of dead microbial colonies. During the saccharification stage, gamma-glutamyltransferase (GT) emerged as the predominant enzyme. Among these enzymes, glycosyltransferase 4 (GT4) presented the highest abundance, followed by glycoside hydrolase family 36 (GH36). As fermentation progressed, the abundance of alpha glucosidase (GH4) increased, and it became the main enzyme. On the 9th day of fermentation, the abundance of this enzyme decreased, indicating a weak and stable fermentation process. Overall, the types of carbohydrate enzymes metabolized by microorganisms constantly changed at different stages of fermentation. In the early stage of fermentation, hydrolytic enzymes are generated mainly to decompose large-molecule carbohydrates. During the middle and late stages of fermentation, enzymes are gradually generated to synthesize small-molecule carbohydrates and other functional components.

Through the analysis of gene annotations for different GHs and GTs across various brewing stages, it was found that different microbial genera contributed significantly to the activity of various GHs (Supplementary Table S1), with bacteria playing a major role. *Weissella* and *Pediococcus* were core functional genera involved in carbohydrate metabolism during the brewing process, contributing to multiple GHs throughout the entire brewing process, including GH1, GH2, GH4, GH13, GH25, and GH32. In contrast, *Klebsiella* and *Exiguobacterium* demonstrated significant gene annotation enrichment specifically during the saccharification and fermentation phases, with predominant functional associations with the glycoside hydrolase families GH13, GH25, and GH32. *Lactobacillus* presented the highest annotation dominance for GH4, GH36, and GT4, indicating its pivotal involvement in both carbohydrate metabolic pathways and glycosyl transfer mechanisms. Moreover, *Ogataea* was the primary contributor to GH28, highlighting its key role in polygalacturonic acid degradation. Additionally, among fungal genera, *Rhizopus* contributed the most to lactase (GH1) throughout the brewing process, and *Saccharomyces* played an important role in the gene annotations of GH32.

Enzyme-coding genes in potential flavor-producing pathways were taxonomically annotated to identify their species origins. The enzymes and microorganisms associated with flavor formation are detailed in Supplementary Table S2, and a total of 88 enzymes and 48 microbial genera related to these metabolic pathways were identified. The metabolic pathways responsible for the formation of key flavor compounds were predicted (Figure 6G). Glucose and fructose, as the primary substrates in alcoholic fermentation, are converted into pyruvate and acetyl-CoA via glycolysis and subsequent decarboxylation. Pyruvate and acetyl-CoA act as key intermediates in the metabolic network, serving as precursors for the biosynthesis of acids, alcohols, and esters.

## 4 Discussion

The flavor and quality of Baijiu are closely correlated with microbial metabolism and abiotic factors during the fermentation process (Han, et al., 2023). In this study, we focused on RFB fermentation and explored the abiotic and microbial contributions of volatile components during the entire fermentation process.

During grain fermentation, physicochemical indicators (e.g., starch, reducing sugars, total acid, pH) are crucial for flavor formation. In practical production, fermentation efficiency is increased by controlling these indicators within optimal ranges, which regulate microbial growth/metabolism and drive structural shifts in the microbial community (Li et al., 2024). Our study revealed that both acidity and total ester content continuously increased with fermentation progression, whereas the reducing sugar content correspondingly decreased during different brewing stages of RFB (Figure 2A). In the production of RFB, acidity is one of the key determinants of Baijiu quality; appropriate acidity in mash can effectively ensure Baijiu quality by inhibiting the growth of harmful bacteria and the production of harmful substances. In addition, acidity can participate in the esterification process and improve the aroma and taste of Baijiu (Pang et al., 2023). The total ester content is another important indicator of the quality of RFB. Studies have shown that esters can endow Baijiu with fruit, flower and other pleasant flavors (Huang et al., 2018). In our study, we found that the esters with the highest concentrations included ethyl lactate, followed by ethyl hexadecanoate and then ethyl acetate. This result was different from those of other reports, which indicated that ethyl lactate and ethyl acetate were the esters with the highest concentrations. However, Yin et al. (2020) also detected many fatty acid ethyl esters, such as ethyl hexadecanoate and ethyl linoleate, in RFB.

The production of Baijiu is related not only to environmental factors but also to the participation of various microorganisms in fermentation, which is a key factor (Zou et al., 2018). Unlike those of other Baijiu flavor types, the microbial composition of RFB is relatively simple. In this study, the dominant fungal genera were *Saccharomycopsis*, *Saccharomyces*, and *Rhizopus*, and the main bacterial genera were *Lactobacillus*, *Pediococcus*, *Weissella*, and *Limosilactobacillus.* Moreover, there was not much difference in fungal richness and diversity among the samples from *Jiuqu* and mash, whereas the bacterial richness and diversity were greater than those of fungi. Among them, the bacterial richness and diversity were highest in the saccharified samples. Some studies reported similar results (Hu et al., 2021; Wang et al., 2023). Although the diversity of fungi is not as high as that of bacteria, fungi play a key role in the production of the RFB. Studies have shown that starch in raw materials for brewing cannot be directly utilized by most yeasts and bacteria and that *Rhizopus* can produce amylase and saccharifying amylase in the saccharification and fermentation of Baijiu and degrade macromolecular carbohydrate compounds in raw materials into reducing sugars (Wang et al., 2020a). *Saccharomyces*, especially *S. cerevisiae*, is not only the main producer of ethanol but also has an important influence on the synthesis and metabolism of higher alcohols during Baijiu fermentation (Wang et al., 2020b). *Saccharomycopsis*, predominantly represented by *Saccharomycopsis fibuligera*, serves as the dominant fungus in RFB (Figure 3C). According to recent reports, *S. fibuligera* is commonly found in starch-rich environments and serves as the dominant amylolytic yeast involved in traditional fermented food production, including rice- and sorghum-based fermentation systems (Xie et al., 2021a; Xie et al., 2021b; Zhou et al., 2022). The strain could also contribute to the flavor and quality of Baijiu and produce a variety of esters, lactones, alcohols, naphthols, acids, aldehydes and ketones in sorghum culture medium, which can increase the concentrations of ethyl acetate, isoamyl acetate, phenylethanol, phenylethyl acetate, and ethyl palmitate in solid fermentation products (Son et al., 2018). Furthermore, the four dominant bacterial genera *Lactobacillus*, *Pediococcus*, *Weissella*, and *Limosilactobacillus* in the present study were all lactic acid bacteria; among them, *Pediococcus* was the dominant genus in *Jiuqu*, and *Lactobacillus* rapidly became the dominant genus during the saccharification and fermentation stages. A study reported that lactic acid bacteria exhibited a growth pH range of 3.0–6.0 with strong acid resistance; could use dextrin to produce lactic acid, ethyl lactate and other flavor components; increased the complexity of the mouthfeel of RFB; and could still reproduce in large numbers in the middle and later stages of fermentation (Hong, et al., 2016). This was also why the acidity of the fermented mash continued to increase throughout the fermentation process. In particular, *Lactobacillus* extensively participates in various metabolic activities, providing functional support and competitive advantages because of its absolute dominance in the later stages of fermentation (Tu, et al., 2022). In addition, carbohydrate metabolites of lactic acid bacteria, such as pyruvic acid, succinic acid and galactose, can promote the growth of yeast. In turn, amino acids synthesized by yeast can support the growth of lactic acid bacteria (Chang et al., 2022). The synergistic effect of yeasts and lactic acid bacteria promoted their dominance as microorganisms in the fermentation of RFB.

Correlation analysis between microorganisms and flavor compounds revealed that the abundances of most fungal and bacterial genera were positively correlated with the dominant flavor components in RFB (Figure 5D). *Mucor* and *Rhizopus* were positively related to ethanol, whereas *Aspergillus*, *Zygosaccharomyces,* and *Pichia* were positively related to ethyl acetate, ethyl (S)-2-hydroxypropanoate, and acetic acid; *Lichtheimia* was positively related to 2-phenylethyl alcohol, 3-methylbutanoic acid, and 2-methylpropanoic acid; and *Pediococcus* was positively correlated with 3-methylbutanoic acid and 2-phenylethyl alcohol. These findings indicate that these microorganisms play important roles in the flavor formation of RFB. Moreover, previous studies have reported that *Mucor* and *Rhizopus* species can convert starch-based substrates into ethanol, with ethanol yields and productivities even higher than those of most known ethanol-producing microorganisms, such as *S. cerevisiae*, *Zygosaccharomyces,* and *Pichia*, whereas *Lichtheimia* and *Pediococcus* are potential producers of 2-phenylethanol, 2,3-butanediol and some acids (Xiang et al., 2019; Chen et al., 2023; Li et al., 2023).

The annotation results of the KEGG functional genes revealed that metabolism was the most annotated functional category, and its content increased from *Jiuqu* to the fermentation stage, reaching the highest abundance on the third day of fermentation (Figure 6A). Among them, carbohydrate metabolism and amino acid metabolism occupied the main pathways (Figure 6B), which was similar to the results of Liu et al.’s research on fermented foods (Liu et al., 2019). Carbohydrates are the main components and energy sources of cellular structure, regulating cellular life activities and providing a material basis for other metabolic processes (Xie et al., 2021a). Furthermore, replication and repair pathways (ko03030, ko03420, ko03430) were also relatively abundant in *Jiuqu* (Figure 6F). These findings indicated that microbial metabolic activity was greater in *Jiuqu* than in the other samples and provided the material basis for the flavor and quality of Baijiu. Previous studies reported that *Jiuqu* fermentation provided approximately 10%–20% of the bacterial community and 60%–80% of the fungal community (Wang et al., 2017). Additionally, the biosynthesis of valine, leucine, and isoleucine (ko00290) was the dominant metabolic pathway in the saccharification stage, whereas C5 branched dibasic acid metabolism (ko00660) and starch and sucrose metabolism (ko00500) were the main metabolic pathways in the fermentation stage (Figure 6C). These findings indicate that these three metabolic pathways play important roles in the formation of flavor substances in RFB.

We identified bacteria, particularly lactic acid bacteria (LAB), such as *Pediococcus* and *Weissella*, as key contributors to various metabolic pathways in RFB. *Pediococcus* emerged as the primary bacterial genus driving ko00290 (valine, leucine, and isoleucine biosynthesis) and ko00660 (C5-branched dibasic acid metabolism) during fermentation. *Pediococcus* and *Weissella* predominantly mediated ko00500 (starch and sucrose metabolism) in *Jiuqu*. During the saccharification and fermentation stages, additional genera, such as *Klebsiella*, *Lactobacillus*, *Unclassified_f_Enterobacteriaceae*, *Citrobacter*, *Cyberlindnera*, *Streptococcus*, *Betabacterium*, and *Enterobacter,* were involved in starch and sucrose metabolism (Supplementary Table S2).

In the metabolic pathway diagram of key flavor compounds in RFB, the ko00500 pathway participated in glycogen degradation, converting glycogen into Glu-6P (glucose-6-phosphate) (Figure 6G). This compound is further broken down via the glycolytic pathway and pyruvate metabolism into pyruvate and acetyl-CoA. As intermediate metabolites, pyruvate and acetyl-CoA play crucial roles in the flavor metabolic network adn participate in the synthesis of various flavor compounds (Li, et al., 2023). Genera such as *Saccharomyces*, *Enterobacter*, *Unclassified_Gammaproteobacteria*, *Klebsiella*, and *Acetobacter* utilize aldehyde dehydrogenase (EC 1.2.1.3) to oxidize acetaldehyde into acetate. Acetate or pyruvate can also be converted back to acetaldehyde. Moreover, *Pseudomonas*, *Unclassified_Proteobacteria*, and S*accharomyces* employ alcohol dehydrogenase (EC 1.1.1.2) to convert acetaldehyde into ethanol, another abundant metabolite in the system. In terms of amino acid metabolism, pyruvate is an essential precursor for amino acid biosynthesis. Pyruvate is utilized to produce leucine, valine, and isoleucine through the enzymatic actions of acetolactate synthase (EC 2.2.1.6) and the branched-chain amino acid transaminase (EC 2.6.1.42), respectively. Moreover, *Pediococcus* and *Weissella* are the primary genera associated with the production of these two enzymes. The metabolic pathways of valine, isoleucine, and leucine are associated with the biosynthesis of higher alcohols (e.g., n-propanol, isobutanol, isoamyl alcohol, and β-phenylethanol) (Xu et al., 2022). As major byproducts of RFB fermentation, higher alcohols exhibit distinct flavor characteristics and are present at elevated concentrations (Wei et al., 2020). Their biosynthesis primarily involves amino acid degradation (Ehrlich pathway) and pyruvate metabolism (Harris pathway) (Ma et al., 2017). In this study, higher alcohol production was predominantly linked to the Harris pathway: pyruvate generated via glycolysis enters branched-chain amino acid biosynthesis (with the isoleucine‒leucine‒valine pathway being central) to form α-keto acids, which are subsequently converted to higher alcohols. Transaminases, decarboxylases, and alcohol dehydrogenases are key enzymes in these pathways, with *Pediococcus*, *Weissella*, *Limosilactobacillus*, *Pseudomonas*, *Unclassified_p_Proteobacteria*, *Saccharomyces*, and *Klebsiella* identified as major contributors to the genes encoding these enzymes (Supplementary Table S2). Additionally, phenylethyl alcohol is a primary flavor compound in RFB, and it can be synthesized through two pathways: the glucose anabolic pathway or amino acid biosynthetic pathways involving valine, leucine, isoleucine, and phenylalanine (Gong et al., 2024). *Saccharomyces* and *Unclassified_f_Saccharomycetaceae* are key microbial taxa harbouring genes for phenylpyruvate decarboxylase (EC 4.1.1.43) in phenylethyl alcohol metabolism, whereas *Pediococcus*, *Klebsiella*, *Weissella*, and *Latilactobacillus* are associated with aryl alcohol dehydrogenase (EC 1.1.1.90) genes. Another common polyol in RFB fermentation, 2,3-butanediol, is generated through the ko00290 pathway, where palmitic acid is metabolized into the intermediates 2-acetolactate and 3-hydroxybutanone. These intermediates are further converted via the ko00650 pathway (butanoate metabolism) (Guo et al., 2023), with contributions from *Weissella*, *Saccharomyces*, *Enterococcus*, *Limosilactobacillus*, *Acetobacter*, and *Pediococcus*.

## 5 Conclusion

In this study, gas chromatography–mass spectrometry (GC‒MS) and metagenomic sequencing were used to detect changes in volatile flavor compounds and microbial community structure in the fermented grains of continuously fermented rice-flavor Baijiu. A total of 84 volatile flavor compounds were detected, with alcohols accounting for the highest proportion, followed by esters and acids. The contents of 2-phenylethanol, ethanol, and isoamyl alcohol were relatively high. The esters were mainly composed of ethyl lactate and ethyl acetate, whereas the acids were mainly composed of acetic acid. The solid saccharification and semisolid fermentation of rice-flavor Baijiu has led to the relatively simple structure of its microbial genera. The dominant fungi were *Saccharomycopsis*, *Saccharomyces* and *Rhizopus.* The dominant bacterial genera included *Lactobacillus*, *Pediococcus*, *Weissella*, and *Limosilactobacillus*. The bacterial diversity was greater than that of fungi during saccharification and fermentation. The correlation network of the main microorganisms and volatile flavor substances indicated that core microbial groups drove the biosynthesis of diverse flavor compounds, and the formation of key flavor components required the collaborative synthesis of multiple microorganisms. Among them, the six core flavor-contributing genera *(Lichtheimia, Kluyveromyces, Lacticaseibacillus, Lactobacillus, Limosilactobacillus, and Schleiferilactobacillus)* exhibit a synergistic metabolic pattern throughout fermentation. Moreover, *Pediococcus* and *Weissella* drive key metabolic pathways, valine/leucine/isoleucine biosynthesis (ko00290) and C5 branched dicarboxylic acid metabolism (ko00660), and the increased alcohol production in RFB is related mainly to the Harris pathway (pyruvate driven) rather than the typical Ehrlich pathway. This study investigated the microbial ecology in both fermentation environments and processes of RFB, which not only provides valuable insights into the holistic ecology of Baijiu fermentation systems but also offers enlightening prospects for quality control in RFB production.

## Data Availability

The datasets presented in this study can be found in online repositories. The names of the repository/repositories and accession number(s) can be found below: https://www.ncbi.nlm.nih.gov/, PRJNA1269740.
